# Diagnostic Performance of Autofluorescence for Oral Lesions: A Comparison Between a Postgraduate and an Expert Clinician

**DOI:** 10.3390/dj13110512

**Published:** 2025-11-03

**Authors:** Alessandro Antonelli, Cristina D’Antonio, Anna Martina Battaglia, Riccardo Finamore, Antonio Madonna, Vincenzo Greco, Vincenzo Cosentino, Selene Barone, Flavia Biamonte, Amerigo Giudice, Francesco Bennardo

**Affiliations:** 1 Department of Health Sciences, School of Dentistry, Magna Graecia University of Catanzaro, 88100 Catanzaro, Italy; alessandro.antonelli@unicz.it (A.A.); cristina.dantonio@studenti.unicz.it (C.D.); riccardo.finamore@studenti.unicz.it (R.F.); antonio.madonna@studenti.unicz.it (A.M.); vincenzo.greco001@studenti.unicz.it (V.G.); vincenzo.cosentino@studenti.unicz.it (V.C.); a.giudice@unicz.it (A.G.); francesco.bennardo@unicz.it (F.B.); 2 Department of Experimental and Clinical Medicine, Magna Graecia University of Catanzaro, 88100 Catanzaro, Italy; am.battaglia@unicz.it (A.M.B.); flavia.biamonte@unicz.it (F.B.)

**Keywords:** oral potentially malignant disorders, mouth neoplasm, dysplasia, fluorescence, VELscope, diagnosis

## Abstract

**Background/Objectives**: Autofluorescence (AF) is a widely used adjunctive tool in the detection of oral potentially malignant disorders (OPMDs) and malignant lesions, but its performance can be influenced by clinicians’ experiences. This study aimed to examine how AF influences diagnostic decision-making and performances of a novice clinician compared with those of an experienced examiner. **Methods**: A total of 80 patients with oral lesions participated in this cross-sectional study. Each underwent a standard oral examination (OE) followed by an assessment with the VELscope^®^ System Vx (LED Medical Diagnostics Inc., Burnaby, BC, Canada), independently conducted by an expert clinician (E) and a postgraduate dentist (PD), both blinded to each other’s results. Biopsy and histopathological analysis provided the reference diagnosis. After every examination, lesions were categorized as either “Risk of Malignancy” (RM) or “No Risk of Malignancy” (NRM). **Results**: Based on OE, PD identified 39 RM lesions, while E 29. AF with VELscope^®^ identified an additional 12 RM lesions for the PD and 7 for the E that were not suspected on OE alone. Combining OE with VELscope^®^ improved sensitivity (PD: 90.9%; E: 95.4%) and negative predictive value (PD: 91.7%; E: 97.6%), while decreasing specificity (PD: 37.9%; E: 70.7%) and positive predictive value (PD: 35.7%; E: 55.3%) compared with OE alone. **Conclusions**: AF increases diagnostic sensitivity, particularly for less experienced clinicians, while offering moderate advantages for experts. Nevertheless, the corresponding decline in specificity emphasizes the need for cautious interpretation. AF should be incorporated as a complementary tool within structured diagnostic pathways, accompanied by adequate training, and cannot replace histopathological confirmation or clinical expertise.

## 1. Introduction

Oral cancer is a major public health concern, ranking as the sixteenth most common malignancy worldwide and the fifteenth in cancer-related mortality (Global Cancer Observatory, 2022) [1]. This is also related to the fact that, unfortunately, nearly half of oral cancers are detected at an advanced stage (III–IV). However, most of them develop from oral potentially malignant disorders (OPMDs) that may persist for several years before progressing [2]. Therefore, early recognition of OPMDs is crucial to improve prognosis, reduce mortality, and minimize treatment-related morbidity [3].

Among the factors contributing to diagnostic delay, professional delay plays a key role. It is influenced by clinicians’ expertise in interpreting patients’ signs and symptoms and by their level of clinical experience [4,5,6,7]. Evidence from studies carried out on students or general practitioners shows a clear link between insufficient knowledge and the inability to apply standardized preventive and diagnostic measures [8,9,10].

Studies have suggested that improved knowledge and continuous training, beginning at the undergraduate level and extending into professional practice, are essential for minimizing diagnostic delays [11,12,13,14]; the visual Oral Examination (OE) is the primary screening method in dental practice employed by both general dental practitioners and Expert clinicians (E) [15,16], and biopsy followed by histological exam remains the gold standard to distinguish OPMDs and malignant lesions from benign ones [17]. What is also important is the development of supportive diagnostic tools that can assist general dentists and specialists in assessing persistent lesions of uncertain biological behavior [18,19,20]. A number of adjunctive diagnostic techniques to support clinical decision-making have been introduced alongside OE to improve early detection of OPMDs [17,21], including vital staining, oral cytology, light-based technique, evaluation of mRNA and protein biomarkers, and emerging artificial intelligence-based systems [17,22,23].

Among light-based tools, autofluorescence (AF) has gained particular interest because it provides non-invasive, real-time visualization of mucosal changes. It is based on the interaction of a high-intensity blue light source (typically 400–600 nm) with endogenous fluorophores of oral mucosal lesions (such as, collagen, NADH, FAD, elastin, keratin, and hemoglobin), which produce tissue-specific fluorescence emission patterns [24,25]. Healthy oral mucosa displays a uniform pale green fluorescence under AF devices, whereas dysplastic or malignant changes often show disrupted stromal collagen and altered fluorophore concentration, leading to a Loss of Autofluorescence (LAF) or to an Alteration of Autofluorescence (AAF), with a darker appearance [26]. However, inflammatory or certain benign conditions can also produce AF patterns due to vascular or metabolic changes, potentially generating false positive (FP) results. A recent systematic review on AF methods has demonstrated a sensitivity for AF > 70%, with an AUC ranging from 0.80 to 0.90, suggesting good diagnostic accuracy [27].

The Visually Enhanced Lesion Scope (VELscope^®^), developed by LED Medical Diagnostics Inc. in collaboration with the British Columbia Cancer Agency, is one of the most widely used chairside AF devices. It consists of a handheld scope emitting 400–460 nm wavelength light to enable direct visualization of oral mucosa under fluorescence. Owing to its handling properties, this device has been extensively adopted in clinical settings, and its diagnostic performances have been widely evaluated in the literature. A systematic review of 25 studies reported an average sensitivity of 70.19% (range: 22–100%) and average specificity of 65.95% (range: 8.4–100%), along with a high number of FP results. Therefore, although VELscope^®^ represents an excellent tool for the detection of oral mucosal lesions, it lacks the ability to differentiate between benign, malignant or inflamed areas. Consequently, its effective use requires the clinical experience and judgment of a trained clinician [28].

Despite this, relatively few studies have addressed the role of clinician experience in diagnostic accuracy and biopsy decision-making when AF is used in conjunction with OE [29,30]. It remains unclear to what extent AF may mitigate the limitations of clinical inexperience in recognizing high-risk lesions or in guiding biopsy site selection. Clarifying these dynamics is important to define training requirements, optimize referral pathways, and balance the risks of unnecessary biopsies against the danger of missing high-grade lesions.

Accordingly, the present study aims to assess the influence of clinician experience (postgraduate dentist vs. expert) on diagnostic parameters in detecting OPMDs and malignancies using OE alone, AF alone, and a combined approach. A secondary objective is to evaluate how AF influences biopsy decision-making—specifically, whether AF-guided selection of biopsy sites differs between generalists and experts. By enrolling both clinically suspicious and benign lesions, we also evaluate each clinician’s ability and VELscope^®^’s diagnostic capacity to distinguish across a broad lesion spectrum. Based on the previous literature, we hypothesize that AF improves sensitivity in both groups, with a more pronounced reduction in specificity among less-experienced clinicians, while experts can integrate AF findings more effectively in guiding biopsy decisions.

## 2. Materials and Methods

This cross-sectional study was conducted at the Dentistry Department of the University “Magna Graecia” of Catanzaro between March 2019 and March 2024. The methodology conformed to the Strengthening the Reporting of Observational Studies in Epidemiology (STROBE) guidelines [31]. This study was conducted in accordance with the Declaration of Helsinki and approved by the Institutional Review Board of Regional Ethical Review Board of Central Calabria (reference for the Magna Graecia University of Catanzaro) (17 January 2019 Nr. 24/2019).

### 2.1. Study Sample

**Figure 1:**
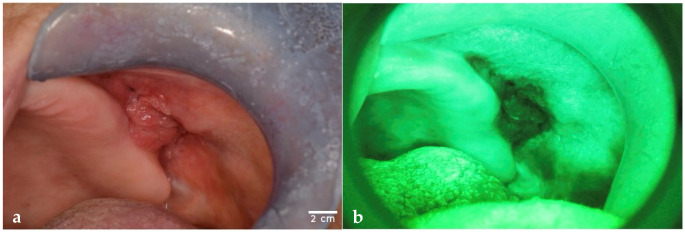

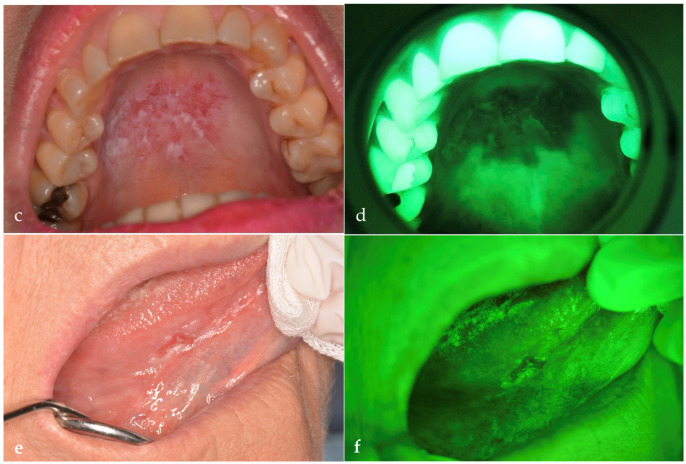
The images show lesions observed with OE and with VELscope^®^ (AF). (**a**) OSCC observed on OE and (**b**) on AF. (**c**) Erosive OLP observed on OE and (**d**) on AF. (**e**) Capillary subepithelial hemangioma observed on OE and (**f**) on AF. This lesion was misdiagnosed as an erythroplakia by both PD and E. Images were obtained with a Nikon D5200 and AF Micro-Nikkor 105 mm lens (1:1 magnification). Scale bar = 2 cm is the same for each picture and has been drawn with ImageJ v0.6.0.

A total of 80 patients presenting consecutively as first visits for oral lesions were enrolled in the study (Figure 1). All patients were informed about the purpose of the study and provided written informed consent.

Subjects included in the study were aged 18 years or older and presented at least one lesion in the oral cavity persistent for more than 14 days.

The exclusion criteria were as follows:

- Pregnant women;
- Patients with pre-treated lesions (laser treatment, medical treatment, surgery);
- Patients with bioptic examination already performed.

### 2.2. Data Collection

Socio-demographic data, including age, gender, smoking and drinking habits, systemic comorbidities, and current medication usage, were recorded.

Every patient on the same day underwent a standardized diagnostic protocol conducted independently by both a postgraduate dentist (PD) and an expert (E). The PD had 3 years of experience after graduating but never completed a postgraduate training course in oral medicine and pathology; the E was, instead, an expert in the field of oral medicine and oral surgery.

First, every patient underwent a conventional visual and manual OE under incandescent operative white light. For each lesion, clinical details—including margins, color, location, surface appearance, and phenotype—were recorded. Standardized photographic documentation was obtained (Figure 1) using a Nikon D5200 camera (AF Micro Nikkor 105 mm, Nikon, Tokyo, Japan) set with the following parameters for intra-oral photographing: manual program mode, shutter speed 1/125 s, fixed focus F32, and magnification ratio 1:1.

After, both PD and E, blinded to each other’s evaluations, independently classified each lesion as either Non-Risk of Malignancy (NRM) or Risk of Malignancy (RM) [2,32,33]. This assessment was based on predefined clinical indicators: non-homogeneous appearance (e.g., mixed erythematous and keratotic patterns such as speckled or erosive lesions), non-uniform morphology, large size (>200 mm^2^), and verrucous phenotype—all considered strong predictors of malignancy risk [33,34,35]. Each examiner also recorded biopsy recommendations, proposed biopsy sites, and provided a provisional clinical diagnosis.

Immediately following OE, the same clinicians carried out an AF-guided examination under dimmed ambient lighting, utilizing the VELscope^®^ System Vx (LED Medical Diagnostics Inc., Burnaby, BC, Canada) device, which emits light in the 400–460 nm wavelength range. Patients wore protective eyewear during the procedure. During this evaluation, updated information on lesion features was recorded, with particular attention to fluorescence changes: AAF for modifications in fluorescence patterns and LAF for completely darkened areas suggestive of mucosal alteration. AF findings were documented photographically using the Nikon D5200 camera fitted with a VELscope^®^ adapter (Figure 1). After AF assessment, both clinicians, still blinded to each other, reassessed malignancy risk, biopsy need and location, and diagnostic hypotheses based on fluorescence outcomes.

At the end, all patients underwent either scalpel incisional or excisional biopsy, performed by an experienced oral and maxillofacial surgeon. Excisional biopsy was reserved for small lesions with a benign appearance, according to E’s opinion. In all other cases, an incisional biopsy was preferred [36,37]. Biopsies were taken from the most representative areas, focusing on sites with heterogeneous clinical features or the most marked AF alterations. In cases of multifocal lesions, the most diagnostically relevant site was selected. Specimens were immediately fixed in 10% buffered formalin and submitted for histopathological examination by a pathologist, who was blinded to the AF findings and the clinical assessments of both examiners.

### 2.3. Statistical Analysis

All statistical analyses were performed using R (version 4.3; R Core Team; Vienna, Austria).

A preliminary pilot study on 10 patients was conducted to estimate the required sample size needed for comparing two proportions. Based on the expected difference in RM detection after AF assessment (P1 = 0.9; P2 = 0.7), with significance level α = 0.05 and statistical power β = 0.8, a total of 72 participants was calculated as necessary.

Descriptive statistics were computed for all socio-demographic variables of patients and clinical variables of lesions after OE and AF. Categorical variables, including RM assessments, were reported as absolute frequencies and percentages.

Inter-examiner agreement between E and PD was quantified using Cohen’s kappa coefficient (κ) with 95% confidence intervals (CI). Kappa values were interpreted according to Landis and Koch criteria (κ < 0.00, poor; 0.00–0.20, slight; 0.21–0.40, fair; 0.41–0.60, moderate; 0.61–0.80, substantial; 0.81–1.00, almost perfect) [38].

Diagnostic performance was evaluated by calculating sensitivity, specificity, positive predictive value (PPV), negative predictive value (NPV), and accuracy for each examiner (PD and E) under both examination conditions (OE and AF). As both modalities were independently applied to the same lesions, tests were analyzed in parallel.

Differences in diagnostic performances between AF and OE on the same lesions were assessed using McNemar’s test for paired nominal data. The continuity-corrected χ^2^ statistic was used, with a two-tailed *p*-value < 0.05 considered statistically significant.

A multivariable logistic regression was constructed to explore associations between clinical predictors of the lesions (dishomogeneous phenotype and color categories: keratotic, erythema, keratotic + erythema, pigmented) and the assessed RM, separately for each operator (PD and E) and after each diagnostic modality (OE and AF). Odds ratios (ORs) with 95% CI and *p*-values were calculated. A two-tailed α-level of 0.05 was considered statistically significant.

## 3. Results

### 3.1. Descriptive Analysis

Table 1 presents the sociodemographic characteristics of the study population.

The majority of patients were female (52.5%, n = 42), with a mean age of 54.0 ± 14.6 years.

A total of 51.2% of patients (n = 41) were current smokers (more than 10 cigarettes/day).

A total of 104 oral lesions were identified. As detailed in Table 2, the buccal mucosa was the most common site (48.1%, n = 50), followed by the tongue (25%; n = 26). 

More than half of the lesions displayed a non-homogeneous phenotype (50.9%, n = 53), with a mixed color appearance (45.2%, n = 47) and a flat morphology (84.6%, n = 88).

Out of 104 lesions, 80 underwent scalpel biopsy.

As reported in Table 3, histopathological analysis of the 80 biopsied lesions revealed that 22 (27.5%) presented malignant histopathological features. These included 5 Oral Squamous Cell Carcinomas (OSCCs) (6.2%), 2 carcinomas in situ (2.5%), 1 adenosquamous carcinoma (1.2%), and 13 OPMDs (16.2%). The most frequent OPMD was erosive Oral Lichen Planus (OLP) (11.2%, n = 9), which showed moderate-to-severe dysplasia [39,40,41,42]

Among the 58 non-malignant lesions, the most common were traumatic ulcer (17.5%; n = 14) and keratotic OLP (16.2%, n = 13), which showed no histological evidence of dysplasia.

### 3.2. Malignancy Risk Assessment and Biopsy Indications After OE

After OE alone, both the PD and E independently assessed RM, as summarized in Table 3.

**Figure 2:**
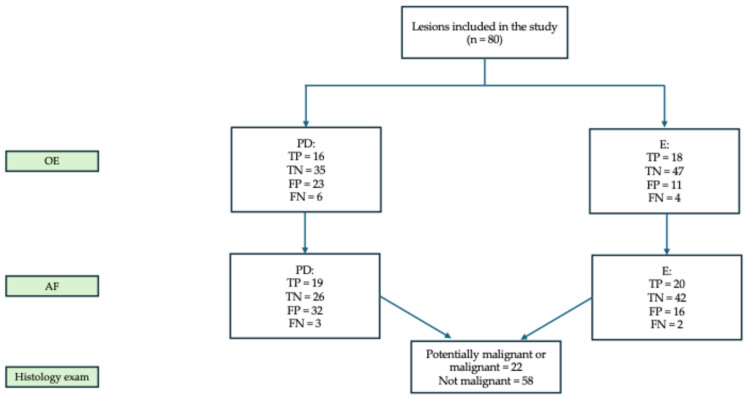
Assessment of RM for each examiner after each examination. Abbreviations: OE = oral examination; PD = postgraduate dentist; E = expert; AF = autofluorescence examination; TP = True Positive; TN = True Negative; FP = false positive; FN = False Negative.

The PD classified 39 lesions (48.7%) as RM, of which 16 were True Positives (TP) and 23 FP. Among the 58 non-malignant lesions, 35 were correctly identified as True Negatives (TN) (Figure 2).

As reported in Table S1, PD recommended biopsy in 70 cases (87.5%), based either on overtly suspicious features, diagnostic uncertainty or on the presence of small benign lesions, such as fibromas. However, the PD did not suggest biopsy for one erosive OLP that later revealed moderate–severe dysplasia, five keratotic OLPs, two traumatic ulcers, and one traumatic keratosis, all of which had been classified as NRM.

The E evaluated 29 lesions (36.2%) as RM, with 18 TP and 11 FP (Figure 1). Biopsy was recommended in 69 cases (86.2%), including for vesciculo-bullous lesions suspected for pemphigus vulgaris, small benign lesions, and cases of diagnostic doubt (Table S1).

Notably, four traumatic lesions (two ulcers and two keratotic lesions) that were considered biopsy-worthy by the general dental practitioner (PD) were recommended for a “wait-and-see” approach (Table S1). Conversely, the E advised biopsy for five keratotic OLP lesions, whereas the PD did not, in order to obtain histological confirmation.

Among malignant cases missed by PD after OE, one adenosquamous carcinoma was misinterpreted as a traumatic ulcer and one mucoepidermoid carcinoma as a pleomorphic adenoma. Four erosive OLPs with moderate dysplasia were underestimated by both examiners.

### 3.3. Malignancy Risk Assessment and Biopsy Indications After AF

With AF examination, the PD identified 45 lesions (56.2%) showing LAF, and an additional 27 with AFA, as illustrated in Table 4. Based on these findings, 51 lesions were classified as RM, adding 12 cases compared to OE alone (Table 3), and 29 lesions as NRM, increasing the FP to 32. Following AF, PD recommended biopsy in 78 cases (97.5%), which turned out to be adenosquamous carcinoma (n = 1), keratotic OLP (n = 5), and traumatic ulcers (n = 2). In 11 cases, AF led to a change in the chosen biopsy site compared with OE alone. These included carcinoma in situ (n = 1), adenosquamous carcinoma (n = 1), erosive OLP (n = 1), and keratotic OLPs (n = 2).

The E identified 41 lesions (51.2%) with LAF and 15 additional lesions (53.8%) with AAF (Table 4). After AF assessment, 36 lesions (45%) were classified as RM, adding 7 cases more than with OE (Table 3), with 16 FP in total. Biopsy was recommended in 49 cases (61.2%). AF also influenced biopsy site selection in two instances: one carcinoma in situ and one keratotic OLP, both showing higher levels of AAF.

### 3.4. Changes in Diagnostic Concordance Between PD and E

As shown in Table S2, the initial clinical diagnostic hypothesis proposed by PD was confirmed by histopathology analysis in 54 cases (67.5%), while E’s diagnostic impressions were validated in 71 cases (88.7%).

The PD misdiagnosed an adenosquamous carcinoma as a traumatic ulcer, and a mucoepidermoid carcinoma as a pleomorphic adenoma; however, both cases were correctly classified as RM following AF assessment. Both examiners misclassified one of the two in situ carcinomas as leukoerythroplakia. Similarly, two erosive OLP lesions were mistaken for leukoerythroplakia due to their plaque-like appearance. A subepithelial capillary hemangioma, presenting as a firm, erythematous lesion with well-defined margins, was misinterpreted as erythroplakia by both examiners (Figure 1e,f). Conversely, a fibroma with surface ulceration and irregularity was erroneously classified as OSCC by both PD and E. All cases of Peripheral Giant Cell Granuloma (PGCG) were falsely considered RM by PD while the E misclassified two such cases, mainly due to a complete LAF pattern. One keratotic OLP, initially assessed as NRM during OE, was reclassified as RM after AF examination by both clinicians because of marked AAF. Another keratotic OLP was misdiagnosed by PD as a melanoma owing to its pigmented features. A melanocytic nevus was incorrectly identified by PD as smoker’s melanosis. Notably, pemphigus vulgaris had the highest rate of diagnostic error by the PD, being variously misdiagnosed as erythroplakia (n = 2), traumatic ulcer (n = 1), and carcinoma in situ (n = 1). Traumatic ulcers were also frequently misclassified: the PD diagnosed one as leukoerythroplakia, one as carcinoma in situ, and three as OSCC; the E misclassified one case as leukoerythroplakia. For traumatic keratosis, the PD misdiagnosed one lesion as carcinoma in situ and two as leukoerythroplakia, with one of these errors shared by the E.

### 3.5. Clinical Predictors of RM Assessment

Table 5 presents the multivariable logistic models predicting RM assessment for each examiner and examination modality. For EO performed by PD, non-homogeneous lesions were significantly more likely to be assessed as malignant or potentially malignant (OR = 3.06; 95% CI 1.17–8.03; *p* = 0.0231). Although erythematous lesions showed increased odds of being classified as malignant (OR = 1.14), this finding did not reach statistical significance. Under AF evaluation by PD, all the clinical variables showed predictive trends, even if without statistical significance. In the case of the E, non-homogeneous color emerged as the strongest predictor of RM, both during OE (OR = 4.3; 95% CI 1.51–12.2; *p* = 0.006) and, to a lesser extent, AF assessment (OR = 2.77; 95% CI 1.02–7.51; *p* = 0.046).

### 3.6. Inter-Operator Agreement for OE and AF

Table 6 presents the inter-rater reliability between PD and E for both OE and AF.

Moderate inter-rater agreement was observed for OE (κ = 0.494), whereas agreement was lower for AF (κ = 0.284), reflecting greater variability in the interpretation of AF findings.

### 3.7. Sensitivity, Specificity, PPV, NPV, and Accuracy

The clinicians’ RM assessment after OE and AF were compared with histopathological findings.

AF increased sensitivity for both clinicians (PD: 86.4%, E: 95.4%), corresponding to gains of 18.2% and 13.6%, respectively, as well as NPV (PD: 89.3%, E: 97.7%). Conversely, specificity decreased when AF was used alone (PD: 43.1%, E: 74.1%). When OE and AF were combined, sensitivity improved further (PD: 90.9%, E: 95.4%) but at the cost of a more pronounced reduction in specificity (PD: 37.9%, E: 70.7%). Diagnostic accuracy was highest for OE alone, both for the PD (62.5% vs. 55%) and the expert (81.25% vs. 80%) (Table 7). According to McNemar’s test with continuity correction, the difference between OE and AF was statistically significant for the PD (χ^2^ = 10.240; *p* = 0.001) but not for the expert (χ^2^ = 1.778; *p* = 0.182) (Table 8).

## 4. Discussion

This study aimed to determine how clinician experience influences detection of OPMDs when AF is used as an adjunct to conventional OE. By including both lesions clinically suspicious for OPMDs and clearly benign lesions, we evaluated AF’s discriminatory value and compared examiners’ diagnostic abilities across a broad spectrum of oral conditions.

Leuci and Coppola et al. (2020) [29] conducted a comparable study in dentists who followed an oral medicine training program versus general dentists and observed that experienced clinicians benefitted more from VELscope^®^ use than general dentists, suggesting that AF’s value for less-experienced practitioners depends on structured training.

Instead, Simonato et al. (2017) [30] showed that fluorescence visualization improved the sensitivity and overall accuracy of inexperienced examiners to a level comparable to that of experts using OE alone.

In our cohort, AF increased sensitivity and NPV for both clinicians, but this gain was accompanied by a reduction in specificity and PPV. The difference between OE and AF reached statistical significance only for the PD (McNemar *p* = 0.001 **), but not for the E, suggesting that less experienced clinicians may rely more heavily on AF findings, sometimes overestimating malignancy risk. From this perspective, our findings partially align with Leuci and Coppola [29], emphasizing the importance of structured training to maximize AF’s utility. At the same time, they slightly diverge from Simonato et al. (2017) [30], who showed an overall diagnostic accuracy for PD using AF to levels comparable to experts using OE alone. While our data also show improved sensitivity for the less-experienced clinician, the overall accuracy of the less experienced-clinician did not fully reach that of the expert, indicating that while AF can enhance sensitivity, it cannot entirely replace clinical judgment.

Dentists play a fundamental role in early recognition and diagnosis of OPMDs and oral cancer, especially in countries where regular dental attendance is common, as opportunistic screening may reduce diagnostic delay [43,44]. Current guidelines designate visual and tactile OE and relative biopsy as the gold standard for early detection of OPMDs [45]. However, multiple studies highlight persistent knowledge gaps among general dental practitioners in recognizing mucosal pathology [46,47,48,49,50,51]. These gaps support the potential role of adjunctive diagnostic tools during OE in clinical decision-making [52,53] but highlight that any adjunct must be interpreted within a clinical context and accompanied by education.

Among light-based tests, like chemiluminescence and AF, the latter has displayed superior accuracy levels in identifying premalignant lesions and early neoplastic changes [54]. Multiple AF devices have been developed through the years (VELscope^®^, approved for oral use in 2008, IllumiScan^®^ [55], GOCCLES^®^ [56,57]).

Systematic reviews, however, have consistently observed limited specificity for AF. Nagi et al. [27] concluded that VELscope^®^ can assist trained clinicians in detecting precursor lesions but cannot reliably differentiate dysplasia from inflammatory conditions. This pattern is reflected in our findings: AF increased FP, particularly for the PD, producing a 15.5% drop in specificity. Many benign entities (e.g., capillary subepithelial hemangioma, PGCG, keratotic OLP, melanocytic nevus, traumatic ulcers) exhibited LAF or AAF and were therefore classified as RM by AF despite being correctly excluded on OE. This phenomenon is attributable to AF alterations caused by non-neoplastic conditions: inflammation, trauma, and vascular changes often increase submucosal hemoglobin content or alter fluorophore concentrations (collagen, NADH, FAD, porphyrins, elastin, keratin), producing AF patterns that mimic dysplasia or carcinoma [26,58].

Our findings mirror meta-analyses reporting pooled AF specificity around 60% [59], which is generally lower than that of clinical examinations and other tools such as toluidine blue staining. AF does not seem reliable for distinguishing benign from dysplastic or malignant lesions [60], as LAF also occurs across benign lesions [61]. Consequently, AF may increase the number of unnecessary biopsies if interpreted in isolation—an effect observed in our study, where the PD recommended biopsy for seven lesions later proven benign, while the E avoided such procedures, highlighting the operator-dependent nature of AF interpretation. The E’s avoidance of unnecessary biopsies indicates that this limitation could potentially be mitigated through structured training, which may help less experienced clinicians to interpret AF findings more accurately and reduce overestimation of malignancy risk.

Several authors have previously warned about AF’s interpretative limitations in primary care. Bhatia et al. [62] noted that the predominance of benign lesions and the frequent LAF of inflammatory conditions may lead to over-referral and harm. Farah et al. [63] emphasized that clinical judgment—not LAF alone—should guide management, and McNamara et al. [64] argued that low AF specificity constrains its routine use in general practice. Our findings corroborate these concerns: AF reduced inter-observer agreement (OE κ = 0.544 to AF κ = 0.284), illustrating substantial operator dependence in fluorescence interpretation. In conclusion, the tendency of AF to generate FP emphasizes the need for careful contextual interpretation, especially by inexperienced clinicians, despite the larger gain in sensitivity.

Despite these limitations, AF demonstrated clinically useful advantages. In fact, it has been demonstrated that NPV ranges from 81.1% [65] to approximately 94% [59], implicating that most AF-negative patients do not harbor high-risk lesions after examination, which is comforting for both the dentist and the patient [66].

Across systematic reviews, AF-based screening sensitivities range from 74% to 82%, specificity from 52% to 62%, NPV from 79% to 81% [59,65,67], and AUC around 0.815, suggesting reasonable diagnostic accuracy [59] but limited specificity. It was also demonstrated that for overt neoplastic lesions, AF adds little beyond OE, as also in our study both PD and E detected most frank carcinomas on OE; however, when OE is negative for neoplasm, AF may uncover occult or asymptomatic lesions [68].

In our cohort, AF enabled the PD to detect malignant or high-grade lesions missed on OE (with sensitivity gain of 13.7%), including an infiltrating adenosquamous carcinoma, a mucoepidermoid carcinoma, and several erosive OLPs later shown to harbor moderate-to-severe dysplasia. The E also identified additional dysplastic OLP cases with AF.

AF influenced biopsy site selection—11 changes for the PD and 2 for the E—leading to identification of carcinoma in situ and higher-grade dysplasia that might otherwise have been missed. These observations support AF’s role in guiding selection of the most representative biopsy area and in delineating lesion margins: AF often revealed irregular boundaries beyond those apparent on white light, and AF-defined margins have been reported to improve molecular clearance relative to white-light margins, with potential surgical benefits for OSCC management and recurrence reduction [68,69,70]. AF may also assist in balancing excision of at-risk mucosa against preservation of healthy tissue in conditions such as OLP and in early detection of molecular recurrence [70].

Multivariable analysis in our sample identified non-homogeneous appearance as the most consistent clinical predictor of perceived malignancy across both examiners and modalities—reinforcing the clinical value of heterogeneous texture and color as red flags.

Interestingly, we observed a diagnostic concordance in 54 cases (67.5%) for PD and in 71 cases (88.7%) for E. The greatest diagnostic challenge for the inexperienced examiner was pemphigus vulgaris, which was variably misinterpreted as erythroplakia, traumatic ulcer or carcinoma in situ; AF changes did not resolve this ambiguity and in some cases reinforced the diagnostic dilemma. Such diagnostic delays for pemphigus are documented in the literature and reflect the relative under-recognition of mucosal versus cutaneous presentations [71,72,73].

Our findings underscore that AF is a useful tool, as it increases sensitivity, but it yields more false positives in less experienced clinicians, who may lack the specific training to distinguish benign AF alterations (e.g., due to inflammation, pigmentation, denture effects, prior treatments) from true premalignant signals [58]. Therefore, while AF can serve as a useful adjunct, especially in biopsy site decision-making, it cannot replace histopathological examination (which remains the gold standard) and cannot be used as a unique diagnostic device. Clinicians—particularly those with less experience—should interpret AF findings with caution, integrating them with clinical context and lesion history.

This study presents several limitations that need to be acknowledged. This single-center, cross-sectional study involved just one PD and one E examiner. This limited sample of examiners substantially restricts the generalizability of the findings and precludes any robust assessment of interobserver variability. Given that the diagnostic performance of the VELscope^®^ is known to be influenced by the examiner’s clinical experience, as also discussed in this paper, the inclusion of multiple examiners from each professional category would be essential to validate these observations and strengthen external validity. Moreover, the study design did not address long-term outcomes or cost-effectiveness. Larger, multicenter studies with multiple examiners and more heterogeneous populations are necessary to validate these findings and to define optimal training pathways for identification and treatment of oral mucosal pathology.

## 5. Conclusions

AF can improve sensitivity and be a useful adjunct in the detection of OPMDs and oral cancer. However, its limited specificity and operator-dependent interpretation preclude its use as a substitute for OE and histopathology, which remain the diagnostic gold standard. Our results emphasize AF’s value as an adjunctive tool rather than a replacement, highlighting the importance of targeted training in oral medicine and structured AF use.

Strengthening education in oral medicine (including improved training in conventional oral examination) and developing standardized protocols for AF use will help exploit AF’s high NPV while reducing unnecessary biopsies and referrals. Future research should focus on refining AF-based strategies, evaluating combined diagnostic approaches (e.g., AF plus molecular markers or AI), and validating training programs to optimize early detection and management of OPMDs and oral cancers.

## Figures and Tables

**Table 1:** Demographic variables of patients included in the study.

Demographic Variables	Patients (n = 80)
Gender	*Frequency (%)*
Male	38 (47.5)
Female	42 (52.5)
Age (in years)	*Mean ± SD*
	54 ± 14.6
Smoking	*Frequency (%)*
Yes	41(51.2)
No	39 (48.7)

Data are expressed as mean ± standard deviation (SD) for age and as absolute number and percentage for gender and smoking habits.

**Table 2:** Site and characteristics of oral lesions included in the study.

Site and Characteristics of the Lesions	Frequency (%)
*Site*	
Lower lip	2 (1.9)
Buccal mucosa	50 (48.1)
Upper vestibule	1 (1)
Gingiva	6 (5.8)
Tongue	26 (25)
Floor of the mouth	1 (1)
Hard palate	12 (11.5)
Soft palate	5 (4.8)
Tonsillar pillar	1 (1)
*Color*	
Erythema	22 (21.2)
Keratotic	29 (27.9)
Erythema and keratotic	47 (45.2)
Pigmented	6 (5.8)
Appearance	
Flat	88 (84.6)
Exophytic	16 (15.4)
Phenotype	
Homogeneous	51 (49.1)
Dishomogeneous	53 (50.9)

Data are expressed as absolute number and percentage.

**Table 3:** Histological diagnosis of 80 lesions that underwent biopsy and relative malignancy risk assessment after OE and AF for both PD and E.

		PD				E			
		OE		AF		OE		AF	
**Histology**	**Frequency** **(%)**	**RM** **(%)**	**NRM** **(%)**	**RM** **(%)**	**NRM** **(%)**	**RM** **(%)**	**NRM** **(%)**	**RM** **(%)**	**NRM** **(%)**
**Malignant**									
Actinic cheilitis	2 (2.5)	2 (100)	0	2 (100)	0	2 (100)	0	2 (100)	0
Adenosquamous carcinoma	1 (1.2)	0	1 (100)	1 (100)	0	1 (100)	0	1 (100)	0
Mucoepidermoid carcinoma	1 (1.2)	0	1 (100)	1 (100)	0	1 (100)	0	1 (100)	0
OSCC	5 (6.2)	5 (100)	0	5 (100)	0	5 (100)	0	5 (100)	0
Carcinoma in situ	2 (2.5)	2 (100)	0	2 (100)	0	2 (100)	0	2 (100)	0
Leukoplakia	2 (2.5)	2 (100)	0	2 (100)	0	2 (100)	0	2 (100)	0
Erosive OLP	9 (11.2)	5 (55.5)	4 (44.4)	6 (66.6)	3 (33.3)	5 (55.5)	4 (44.4)	7 (77.7)	2 (22.2)
		**PD**				**E**			
		**OE**		**AF**		**OE**		**AF**	
**Not Malignant**	**Frequency** **(%)**	**RM** **(%)**	**NRM** **(%)**	**RM** **(%)**	**NRM** **(%)**	**RM** **(%)**	**NRM** **(%)**	**RM** **(%)**	**NRM** **(%)**
Capillary subepithelial hemangioma	1 (1.2)	1 (100)	0	1 (100)	0	0	1 (100)	1 (100)	0
Fibroma	9 (11.2)	4 (44.4)	5 (55.5)	4 (44.4)	5 (55.9)	1 (11.1)	8 (88.8)	0	9 (100)
PGCG	5 (6.2)	0	5 (100)	5 (100)	0	0	5 (100)	2 (40)	3 (60)
Keratotic OLP	13 (16.2)	8 (61.5)	5 (38.5)	9 (69.2)	4 (30.8)	6 (46.2)	7 (53.8)	7 (53.2)	6 (46.1)
Melanocytic nevus	5 (6.2)	0	5 (100)	1 (20)	4 (80)	0	5 (100)	1 (20)	4 (80)
Pemphigus vulgaris	6 (7.5)	2 (33.3)	4 (66.7)	1 (16.6)	5 (83.3)	0	6 (100)	0	6 (100)
Traumatic keratosis	5 (6.2)	4 (80)	1 (20)	2 (40)	3 (60)	2 (40)	3 (60)	2 (40)	3 (60)
Traumatic ulcer	14 (17.5)	4 (28.6)	10 (71.4)	9 (64.3)	5 (35.7)	2 (14.3)	12 (85.7)	3 (21.5)	11 (78.5)

Abbreviations: PD = Postgraduate Dentist; E = expert clinician; OE = oral examination; AF = autofluorescence examination; RM = Risk of Malignancy; NRM = Non-Risk of Malignancy; OSCC = Oral Squamous Cells Carcinoma; OLP = Oral Lichen Planus; PGCG = Peripheral Giant Cell Granuloma. Data are expressed as absolute number and percentage.

**Table 4:** AF patterns under VELscope^®^ of lesions that underwent biopsy as described by PD and E.

		PD			E		
**Histology**	**Frequency** **(%)**	**Normal** **(%)**	**AAF** **(%)**	**LAF** **(%)**	**Normal** **(%)**	**AAF** **(%)**	**LAF** **(%)**
**Potentially malignant or malignant**							
Actinic cheilitis	2 (2.5)	0	0	2 (100)	0	0	2 (100)
Adenosquamous carcinoma	1 (1.2)	0	0	1 (100)	0	0	1 (100)
Mucoepidermoid carcinoma	1 (1.2)	0	0	1 (100)	0	0	1 (100)
OSCC	5 (6.2)	0	0	5 (100)	0	0	5 (100)
Carcinoma in situ	2 (2.5)	0	1 (50)	1 (50)	0	1 (50)	1 (50)
Leukoplakia	2 (2.5)	0	0	2 (100)	0	0	2 (100)
Erosive OLP	9 (11.2)	3 (33.3)	1 (11.1)	5 (55.5)	2 (22.2)	3 (33.3)	4 (44.4)
		**PD**			**E**		
**Not Malignant**	**Frequency (%)**	**Normal (%)**	**AAF** **(%)**	**LAF** **(%)**	**Normal (%)**	**AAF** **(%)**	**LAF** **(%)**
Capillary subepithelial hemangioma	1 (1.2)	0	0	1 (100)	0	0	1 (100)
Fibroma	9 (11.2)	2 (22.2)	3 (33.3)	4 (44.4)	4 (44.4)	5 (55.5)	0
PGCG	5 (6.2)	0	0	5 (100)	0	3 (60)	2 (40)
Keratotic OLP	13 (16.2)	1 (7.6)	7 (53.8)	6 (46.1)	0	8 (61.5)	6 (46.1)
Melanocytic nevus	5 (6.2)	0	4 (80)	1 (20)	4 (80)	1 (20)	0
Pemphigus vulgaris	6 (7.5)	2 (33.3)	3 (50)	1 (16.6)	4 (66.6)	0	1 (16.6)
Traumatic keratosis	5 (6.2)	1 (20)	2 (40)	2 (40)	3 (60)	2 (40)	0
Traumatic ulcer	14 (17.5)	1 (7.1)	6 (42.8)	8 (57.1)	2 (14.2)	10 (71.4)	1 (7.1)

Abbreviations: PD = postgraduate dentist; E = expert clinician; OE = oral examination; AF = autofluorescence examination; RM = Risk of Malignancy; NRM = Non-Risk of Malignancy; OSCC = Oral Squamous Cells Carcinoma; OLP = Oral Lichen Planus; PGCG = Peripheral Giant Cell Granuloma; AAF = Alterations of Autofluorescence; LAF = Loss of Autofluorescence. Data are expressed as absolute number and percentage.

**Table 5:** Multivariable logistic regression of clinical predictors for RM assessment for every evaluation and for each operator.

	PD				E			
	OE		AF		OE		AF	
**Predictor**	*OR (95% CI)*	*p*	*OR (95% CI)*	*p*	*OR (95% CI)*	*p*	*OR (95% CI)*	*p*
**Dishomogeous** **phenoype**	3.06 (1.170–8.03)	0.0231 *	1.38 (0.502–3.81)	0.530	4.3 (1.51–12.2)	0.006 **	2.77 (1.02–7.51)	0.046 *
**Keratotic**	<0.001 (0-inf) †	0.996	7.80 (0-inf.) †	0.996	<0.001 (0-inf) †	0.994	<0.001 (0-inf) †	0.994
**Keratotic** **+ erythema**	0.930 (0.275–3.14)	0.907	8.16 (0.173–3.85)	0.798	0.947 (0.271–3.30)	0.932	2.02 (0.527–7.74)	0.305
**Pigmented**	<0.001 (0-inf)	0.993	1.24	0.993	0.631 (0.047–8.52)	0.729	0.723 (0.05–11.30)	0.8170
**Erythema**	1.14 (0.312–4.15)	0.846	4.96 (0.092–2.68)	0.415	0.469 (0.12–1.83)	0.276	0.585 (0.13–2.64)	0.487

Abbreviations: PD = postgraduate dentist; E = expert clinician; OE = oral examination; AF = autofluorescence examination. * Significance: *p* ≤ 0.05. ** Strongly significant *p* ≤ 0.01. † OR (5% CI) values reported as (0–inf) indicate complete separation in the data, where the outcome occurred in only one group. These values reflect limitations of standard OR estimation and should be interpreted accordingly.

**Table 6:** Inter-observer concordance (Cohen’s *κ*) for OE and AF.

Examination	κ (CI 95%)
EO	0.494 (0.303–0.686)
AF	0.284 (0.081–0.4856)

Abbreviations: PD = postgraduate dentist; E = expert clinician; OE = oral examination; AF = autofluorescence examination. Data are expressed as Cohen’s *κ* coefficient with 95% confidence intervals.

**Table 7:** Sensitivity, specificity, PPV, NPV, and accuracy for OE and AF examinations for both PD and E.

PD	OE	AF	Combined
**Sensitivity**	68.2%	86.4%	90.9%
**Specificity**	60.3%	43.1%	37.9%
**PPV**	39.5%	36.5%	35.7%
**NPV**	83.3%	89.3%	91.7%
**Accuracy**	62.5%	55%	52.5%
**E**	**EO**	**AF**	**Combined**
**Sensitivity**	81.8%	95.4%	95.4%
**Specificity**	79.3%	74.1%	70.7%
**PPV**	60%	58.3%	55.3%
**NPV**	92%	97.7%	97.6%
**Accuracy**	81.25%	80%	77.1%

Abbreviations: PD = postgraduate dentist; E = expert clinician; OE = oral examination; AF = autofluorescence examination; PPV = positive predictive value; NPV = negative predictive value. Data are expressed as percentages.

**Table 8:** Differences in diagnostic performance between OE and AF in both PD and E.

Operator	χ^2^ (Continuity-Corrected)	*p*-Value
PD	10.240	0.001 **
E	1.778	0.182

Abbreviations: PD = postgraduate dentist; E = expert clinician; OE = oral examination; AF = autofluorescence examination. A significant difference between the examinations for each operator was measured by McNemar’s test. ** Strongly significant *p* ≤ 0.01.

## Data Availability

The original contributions presented in this study are included in the article. Further inquiries can be directed to the corresponding author.

## Supplementary Materials

The following supporting information can be downloaded at: https://www.mdpi.com/article/10.3390/dj13110512/s1: Table S1: Biopsy indications after OE and AF for both PD and comparison of biopsy indication site between OE and AF for both PD and E.; Table S2: Comparison of diagnostic concordance between PD and E after the histological evaluation.

## Abbreviations

The following abbreviations are used in this manuscript:

AAF	Alterations of Autofluorescence
AF	Autofluorescence
CI	Confidence Intervals
E	Expert
FAD	Flavin Adenine Dinucleotide
FN	False Negative
FP	False Positive
LAF	Loss of Autofluorescence
NADH	Nicotinamide Adenine Dinucleotide
NPV	Negative Predictive Value
NRM	Non-Risk of Malignancy
OE	Oral Examination
OLP	Oral Lichen Planus
OPMDs	Oral Potentially Malignant Disorders
OSCC	Oral Squamous Cell Carcinoma
PD	Postgraduate Dentist
PGCG	Peripheral Giant Cell Granuloma
PPV	Positive Predictive Value
RM	Risk of Malignancy
SD	Standard Deviation
TN	True Negative
TP	True Positive
