# Supplementary material for: Diagnostic Performance of Autofluorescence for Oral Lesions: A Comparison Between a Postgraduate and an Expert Clinician
Source: Dent J (Basel). 2025 Nov 3;13(11):512. doi: 10.3390/dj13110512 (PMC12651155; doi:10.3390/dj13110512)
Supplement: Supplementary file 1 [file dentistry-13-00512-s001.zip › dentistry-3907980-supplementary.pdf]

Table S1. Biopsy indications after OE and AF for both PD and comparison of biopsy indication site between OE and AF for both PD and E.

		PD			E		
		OE	AF		OE	AF	
**Histology**	**Frequency (%)**	**Biopsy**	**Biopsy**	**Biopsy site confirmed**	**Biopsy**	**Biopsy**	**Biopsy site confirmed**
**Potentially malignant or malignant**							
Actinic cheilitis	2 (2.5)	2 (100)	2 (100)	2 (100)	2 (100)	2 (100)	2 (100)
Adenosquamous carcinoma	1 (1.2)	0	1 (100)	0	1 (100)	1 (100)	1 (100)
Mucoepidermoid carcinoma	1 (1.2)	1 (100)	1 (100)	1 (100)	1 (100)	1 (100)	1 (100)
OSCC	5 (6.2)	5 (100)	5 (100)	5 (100)	5 (100)	5 (100)	5 (100)
Carcinoma in situ	2 (2.5)	2 (100)	2 (100)	1 (50)	2 (100)	2 (100)	1 (50)
Leukoplakia	2 (2.5)	2 (100)	2 (100)	2 (100)	2 (100)	2 (100)	2 (100)
Erosive OLP	9 (11.2)	8 (88.8)	8 (88.8)	8 (88.8)	5 (55.5)	7 (77.7)	5 (55.5)
**Not Malignant**							
Capillary subepithelial hemangioma	1 (1.2)	1 (100)	1 (100)	1 (100)	1 (100)	1 (100)	1 (100)
Fibroma	9 (11.2)	9 (100)	9 (100)	9 (100)	9 (100)	9 (100)	9 (100)
PGCG	5 (6.2)	5 (100)	5 (100)	5 (100)	5 (100)	5 (100)	5 (100)
Keratotic OLP	13 (16.2)	8 (61.5)	13 (100)	8 (61.5)	13 (100)	13 (100)	12 (92.3)
Melanocytic nevus	5 (6.2)	5 (100)	5 (100)	5 (100)	5 (100)	5 (100)	5 (100)
Pemphigus vulgaris	6 (7.5)	6 (100)	6 (100)	6 (100)	6 (100)	6 (100)	6 (100)
Traumatic keratosis	5 (6.2)	4 (80)	4 (80)	4 (80)	2 (40)	2 (40)	2 (40)
Traumatic ulcer	14 (17.5)	12 (85.7)	14 (100)	12 (85.7)	10 (71.4)	10 (71.4)	10 (71.4)

Abbreviations: PD = Post-graduate Dentist; E = Expert clinician; OE = Oral Examination; AF = Autofluorescence examination; RM = Risk of Malignancy; NRM = Non-Risk of Malignancy; OSCC = Oral Squamous Cells Carcinoma; OLP = Oral Lichen Planus; PGCG = Peripheral Giant Cell Granuloma.

Data are expressed as absolute number and percentage.

Table S2. Comparison of diagnostic concordance between PD and E after the histological evaluation.

		Diagnostic concordance	
		PD	E
**Histology**	**Frequency (%)**	**Frequency (%)**	**Frequency (%)**
**Potentially malignant or malignant**			
Actinic cheilitis	2 (2.5)	2 (100)	2 (100)
Adenosquamous carcinoma	1 (1.2)	0	1 (100)
Mucoepidermoid carcinoma	1 (1.2)	0	1 (100)
OSCC	5 (6.2)	5 (100)	5 (100)
Carcinoma in situ	2 (2.5)	1 (50)	1 (50)
Leukoplakia	2 (2.5)	2 (100)	2 (100)
Erosive OLP	9 (11.2)	7 (77.7)	7 (77.7)
**Not Malignant**			
Capillary subepithelial hemangioma	1 (1.2)	0	0
Fibroma	9 (11.2)	5 (55.5)	8 (88.8)
PGCG	5 (6.2)	4 (80)	4 (80)
Keratotic OLP	13 (16.2)	11 (84.6)	12 (92.3)
Melanocytic nevus	5 (6.2)	4 (80)	5 (100)
Pemphigus vulgaris	6 (7.5)	1 (16.7)	6 (100)
Traumatic keratosis	5 (6.2)	2 (40)	4 (80)
Traumatic ulcer	14 (17.5)	10 (71.4)	13 (92.8)

Abbreviations: PD = Post-graduate Dentist; E = Expert clinician; OE = Oral Examination; AF = Autofluorescence examination; RM = Risk of Malignancy; NRM = Non-Risk of Malignancy; OSCC = Oral Squamous Cells Carcinoma; OLP = Oral Lichen Planus; PGCG = Peripheral Giant Cell Granuloma.

Data are expressed as absolute number and percentage.
